# Single‐stage revision for chronic fistulized infected total knee arthroplasty achieves infection‐free survival comparable to non‐fistulized cases

**DOI:** 10.1002/ksa.70328

**Published:** 2026-02-06

**Authors:** Arthur Barbaret, Joseph Jacquet, Patrice Wong, David Zhu, Florian Collignon, Thomas Oberle, Louis Lajoinie, Clement Ferri, Kamel Rouizi, Gregoire Vaz, Andrea Fernandez, Sibylle Bevilacqua, Olivier Roche

**Affiliations:** ^1^ Centre Chirurgical Emile Gallé Nancy France; ^2^ Service de Chirurgie orthopédique COTA Nancy France; ^3^ Service de maladies infectieuses, CHRU Nancy Vandoeuvre lès Nancy France

**Keywords:** chronic infection, periprosthetic joint infection, single‐stage revision, sinus tract, total knee arthroplasty

## Abstract

**Purpose:**

The presence of a sinus tract in chronic periprosthetic joint infection (PJI) has traditionally been considered a contraindication to single‐stage total knee arthroplasty (TKA) revision. However, an increasing number of surgeons are now performing single‐stage revisions in this context, despite limited comparative data. This study aimed to evaluate whether single‐stage revision outcomes differ between fistulized (TKA/F+) and non‐fistulized (TKA/F–) infected TKAs.

**Method:**

A retrospective case‐control study was conducted at a regional reference centre for bone and joint infections, including all patients who underwent single‐stage TKA revision for chronic infection between January 2013 and June 2023, with a minimum two‐year follow‐up. Patients were grouped according to the presence (TKA/F+) or absence (TKA/F–) of a sinus tract. The primary outcome was infection‐free survival. Surgical procedures, antibiotic protocols and follow‐up schedules were standardized across both groups. Survival analysis was performed using the Kaplan–Meier method and log‐rank test.

**Results:**

A total of 285 patients were included: 47 TKA/F+ and 238 TKA/F–. The TKA/F+ group had significantly older patients, higher body mass index (BMI) and American Society of Anesthesiologists (ASA) scores, and more comorbidities. Polymicrobial infections were more frequent in TKA/F+ (57% vs. 22%). Despite these differences, infection‐free survival rates were similar: 81.2% (±4.3) in TKA/F+ versus 83.6% (±2.2) in TKA/F–, with no statistically significant difference. Surgical management included extensive debridement, systematic sampling, gentamicin‐loaded cement and local flap coverage when needed.

**Conclusion:**

Single‐stage revision may be effective in patients with a sinus tract when performed within a structured surgical and antibiotic protocol. Some stronger studies are needed to assess whether the presence of a sinus tract should not be considered an absolute contraindication. These findings support broader consideration of single‐stage strategies in chronic TKA infections.

**Level of Evidence:**

Level III.

**Abbreviations**
ASA: American Society of Anesthesiologists
BMI: body mass index
CRIOAC: Reference Center for Complex Bone and Joint Infections
CRP: C‐reactive protein
DAIR: debridement antibiotics and implant retention
ISID: International Society for Infectious Diseases
MRSA: methicillin‐resistant *Staphylococcus aureus*
MSIS: Musculoskeletal Infection Society
PJI: periprosthetic joint infection
SFHG: French Hip and Knee Society
TKA: total knee arthroplasty
TKA/F+: fustulized infected total hip arthroplasty
TKA/F−: non‐fustulized infected total hip arthroplasty

## INTRODUCTION

Periprosthetic joint infection (PJI) remains one of the most severe complications following total knee arthroplasty (TKA), leading to substantial morbidity, repeated surgical procedures and major socioeconomic costs [1, 20]. With the projected increase in the number of primary and revision TKAs over the coming decades [10, 23], the burden of PJI is expected to rise accordingly. Although two‐stage exchange has long been considered the reference strategy for chronic infections, accumulating evidence suggests that single‐stage revision may achieve comparable infection control while offering advantages in terms of functional recovery, morbidity and healthcare costs [9, 15, 16, 24].

Despite these encouraging results, indications for single‐stage exchange remain debated, and patient selection criteria vary widely across studies. According to international expert consensus and several guidelines, the presence of a sinus tract is traditionally regarded as a contraindication to single‐stage revision, based on the assumption that it reflects a high bacterial load, poor soft‐tissue envelope and an increased risk of persistent infection [12]. However, this recommendation is largely supported by low‐level evidence and extrapolated from heterogeneous series, often combining hip and knee arthroplasties. More recently, this paradigm has been challenged, and a more individualized, evidence‐based approach to contraindications for single‐stage revision has been advocated [6].

In the field of total hip arthroplasty, several studies have challenged this paradigm by reporting satisfactory infection eradication rates after single‐stage revision in fistulized chronic infections, with long‐term survival comparable to that of non‐fistulized cases [21, 28, 31]. In contrast, data focusing specifically on the knee are scarce, and to date, no comparative study has evaluated whether the presence of a sinus tract truly compromises the outcome of single‐stage revision in chronic infected TKAs. This lack of evidence is clinically relevant, as sinus tracts are frequently encountered in referral centres for complex bone and joint infections and often lead to systematic exclusion of patients from single‐stage strategies.

The primary objective of the present study was therefore to compare infection‐free survival after single‐stage revision TKA in patients with a chronic PJI with a sinus tract (TKA/F+) versus those without a sinus tract (TKA/F−) in a large, unselected cohort treated in a regional reference centre. The secondary objective was to identify independent risk factors for treatment failure, with a particular focus on the impact of polymicrobial infection and host‐related comorbidities.

It was hypothesized that, when performed within a standardized surgical and multidisciplinary antibiotic protocol, the presence of a sinus tract would not be associated with a lower rate of infection eradication after single‐stage revision TKA. Demonstrating comparable outcomes between fistulized and non‐fistulized cases would challenge the concept of the sinus tract as an absolute contraindication and could support a broader, evidence‐based use of single‐stage strategies in chronic knee PJI.

## MATERIALS AND METHOD

### Study design and population

Each included patient was informed, and none returned an objection form within the established timeframe.

All patients who underwent a TKA revision at the Émile Gallé Surgical Centre, Nancy, France, between January 2013 and June 2023, with a follow‐up of at least two years, were eligible. This institution is designated as a Reference Centre for Complex Bone and Joint Infections (CRIOAC).

Inclusion criteria were: age ≥18 years, single‐stage TKA revision for chronic infection and a postoperative antibiotic treatment duration of at least three months. PJI was considered chronic when they occurred 30 days after the index surgery, in accordance with the classification of Tsukayama et al. [26]. Patients with a history of prior PJI treatment were still included. Exclusion criteria were: patients lost to follow‐up before 2 years after surgery, patients who received debridement antibiotics and implant retention (DAIR) or two‐stage replacement. During the study period, bacteriological samples were systematically obtained in all revision procedures performed for loosening. These analyses included direct examination, culture and bacterial DNA testing. Loosening was considered mechanical when all samples returned negative results.

Clinical evaluations were performed at 3 months, 1 year and 2 years postoperatively and then every 2 years. The primary outcome was the absence of infection recurrence. Septic recurrence was defined as a relapse involving the same microorganism, a new osteoarticular infection in the same knee caused by a different pathogen or death related to the studied infection.

The following risk factors for PJI were recorded: sex, age, body mass index (BMI), American Society of Anesthesiologists (ASA) score and diabetes mellitus. With regard to diabetes control, patients with glycated haemoglobin levels above 8.5% were systematically referred for medical hospitalization to achieve adequate metabolic control.

Patients were classified into the ‘case’ group if a sinus tract was present, and into the ‘control’ group if no sinus tract was identified.

The local ethics committee of Centre Hospitalier Universitaire de Nancy (approval number 2024PI232) approved this single‐centre retrospective case‐control study.

Because of the marked imbalance in sample size (TKA/F+ = 47 vs. TKA/F– = 238), the authors performed a post hoc power analysis using a two‐sided two‐proportion comparison (*α* = 0.05). Using the observed infection‐free survival rates at last assessment (81.2% in TKA/F+ and 83.6% in TKA/F–), the power to detect the observed absolute difference of 2.4 percentage points is about 7%, indicating a high risk of Type II error. With the current sample sizes, the minimum detectable difference at 80% power is approximately 21 percentage points. These findings suggest that the non‐significant result may reflect insufficient power rather than strict equivalence between groups. Nevertheless, this cohort originates from a regional referral centre for bone and joint infections over a period of more than ten years. This study remains clinically relevant, given the low proportion of fistulized TKAs among chronic infections.

### Diagnosis of PJI

For this study, PJIs were defined according to the Musculoskeletal Infection Society (MSIS) criteria [18] and documented by intraoperative culture results. A minimum of three separate samples was collected. A microorganism was considered causative if it was isolated from at least two distinct samples. Chronic infection was defined according to Tsukayama Stage IV [25, 26], that is, occurring more than four weeks after prosthesis implantation, with clinical symptoms and positive microbiological cultures. The following comorbidities were recorded from the patients' medical records: follow up, age, sex, side, BMI, ASA score, previous knee surgery and diabetes. The presence of a sinus tract had to be documented either during the preoperative consultation or in the operative report.

The diagnosis and therapeutic strategy were validated during a multidisciplinary expert meeting (CRIOAC), involving at least one orthopaedic surgeon specialized in bone and joint infections, one infectious disease specialist and one microbiologist.

### Surgical procedure

Both the case and control groups underwent the same single‐stage replacement procedure, performed by two senior surgeons trained in single‐stage TKA revision for septic indications. For each procedure, the patient was placed in the supine position, and no tourniquet was used. Sinus tracts were identified under direct visualization, without additional exploration using methylene blue or fluoroscopy. When a sinus tract was present, it was excised. The first step consisted of debridement of the soft tissues and removal of all infected material. The implanted prosthesis, any foreign materials, and all cement were completely removed. At least three samples of tissue were collected (from the femur, tibia, soft tissues, and, when present, intra‐articular fluid) and sent to the microbiology laboratory for direct examination, culture, and antibiotic susceptibility testing. Pulsatile lavage with 3 L of normal saline was performed. An additional sterile drape layer was applied to ensure a clean operative field, followed by reimplantation of a new prosthesis using gentamicin‐loaded cement. Meticulous haemostasis was achieved during the procedure, and no suction drain was placed. If primary skin closure was not possible or deemed unreliable, a flap was performed by a plastic surgeon from the same institution. The types of flaps used included gastrocnemius, soleus, anterolateral thigh flaps, as well as perforator flaps [2, 3, 19]. The type of flap performed was left to the discretion of the plastic surgeon.

### Antibiotherapy

Empirical broad‐spectrum intravenous antibiotic therapy was initiated in accordance with the recommendations of the CRIOAC. No preoperative antibiotics were administered. This empirical therapy was subsequently adjusted based on pathogen identification and antibiotic susceptibility testing [11].

Biological monitoring was conducted to assess C‐reactive protein (CRP) kinetics and ensure the tolerability of the antibiotics.

### Statistical analysis

Statistical analyses were performed using XLSTAT 2023.2.0 (Addinsoft). Normality of continuous variables was assessed using the Shapiro–Wilk test. Continuous variables are presented as mean ± standard deviation or median (interquartile range), as appropriate. Between‐group comparisons were performed using Student's *t* test for normally distributed variables and the Mann–Whitney *U* test otherwise. A two‐sided *p* value ≤ 0.05 was considered statistically significant.

Kaplan–Meier survival analysis was used to estimate infection‐free survival, with treatment failure as the event of interest. Time zero was defined as the date of surgery, and patients without failure at the last follow‐up were censored. Survival curves were compared using the log‐rank test. Follow‐up was truncated at 70 months to minimize bias related to missing data, as the number of patients at risk beyond this time point was insufficient to allow reliable estimates.

A multivariable logistic regression model was built to identify independent predictors of treatment failure. The dependent variable was treatment failure at last follow‐up (yes/no). The main exposure of interest was the presence of a sinus tract (TKA/F+ vs. TKA/F−). Covariates were selected a priori based on clinical relevance and baseline imbalances and included age, BMI, ASA score (≥3 vs. <3), diabetes and polymicrobial infection. Results are reported as adjusted odds ratios with 95% confidence intervals.

## RESULTS

A total of 47 fistulized (TKA/F+) and 238 non‐fistulized (TKA/F–) chronic infected TKAs were included (Figure 1). Baseline characteristics are summarized in Table 1. Patients in the TKA/F+ group were significantly older and presented with higher BMI, higher ASA scores, more frequent diabetes, and a greater history of previous surgery on the affected knee compared with the TKA/F– group.

**Figure 1:**
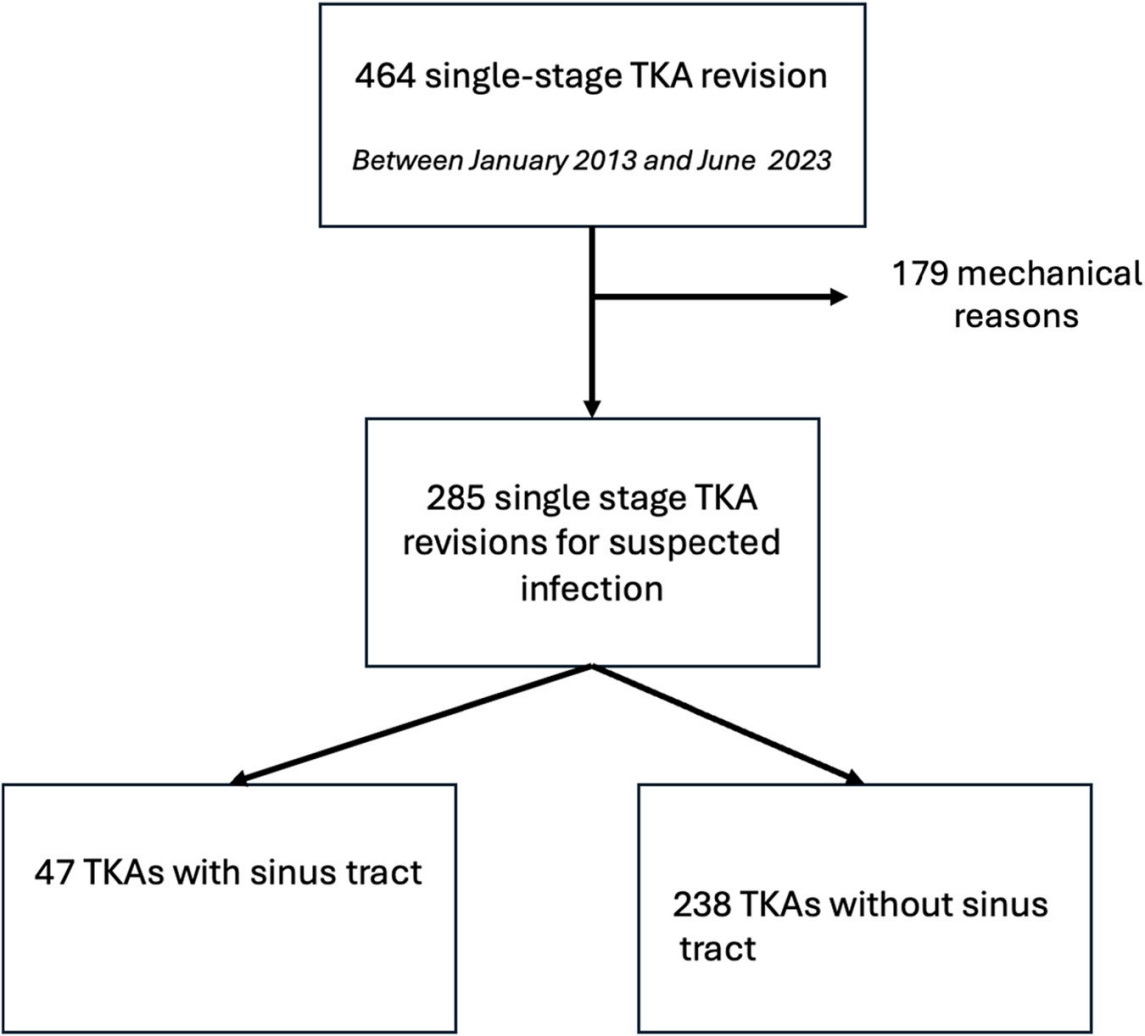
Flowcharts. TKA, total knee arthroplasty.

**Table 1:** Baseline characteristics.

	TKA/F+ *n* = 47	TKA/F– *n* = 238	*p*
Follow‐up (years)	4.7 [2.8–6.6]	5.2 [3.5–6.9]	0.67
Age (years)	70 [62.8–77.2]	67 [61.1–72.9]	0.041
Sex			0.111
Male	32 (68%)	129 (54%)	
Female	15 (32%)	109 (46%)	
Side			0.03
Right	20 (42%)	147 (62%)	
Left	27 (58%)	91 (38%)	
BMI (kg/m^2^)	34.7 [32.7–36.7]	32.8 [29.1–36.5]	0.031
ASA score			0.021
1	0	11 (5%)	
2	9 (19%)	133 (56%)	
3	30 (64%)	88 (37%)	
4	8 (17%)	6 (2%)	
Previous knee surgery			0.025
Yes	9 (19%)	84 (35%)	
No	38 (81%)	154 (65%)	
Diabetes	33 (70%)	111 (47%)	0.029

Abbreviations: ASA, American Society of Anesthesiologists; BMI, body mass index; TKA, total knee arthroplasty.

Microbiological profiles differed markedly between groups (Figures 2 and 3). Polymicrobial infections were significantly more frequent in the TKA/F+ group, whereas monomicrobial infections, particularly caused by *Staphylococcus aureus*, predominated in the TKA/F– group. No fungal infections were identified in the fistulized group, whereas a small proportion was observed in non‐fistulized cases.

**Figure 2:**
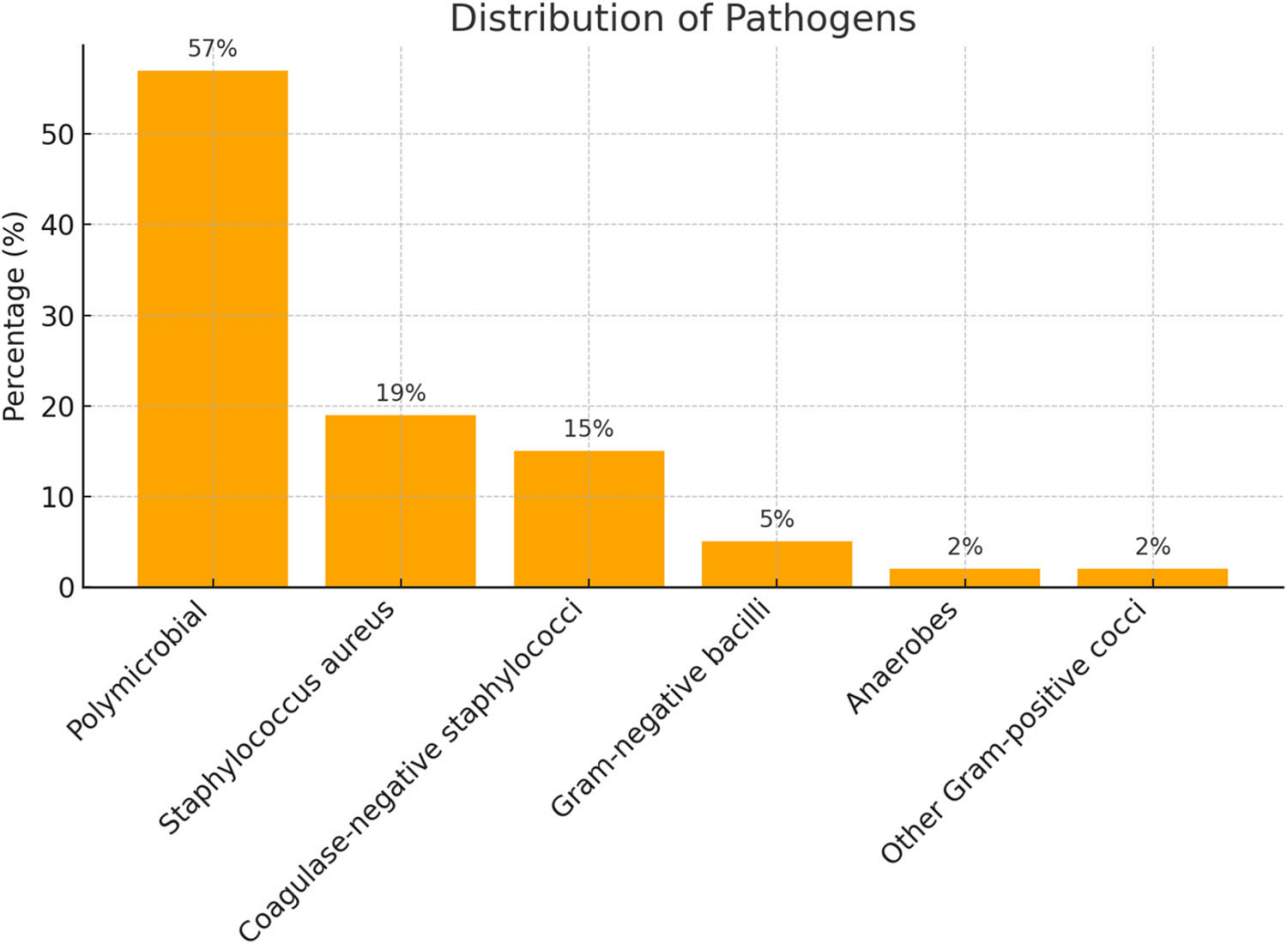
Microbiological results of the TKA/F+ group. TKA, total knee arthroplasty.

**Figure 3:**
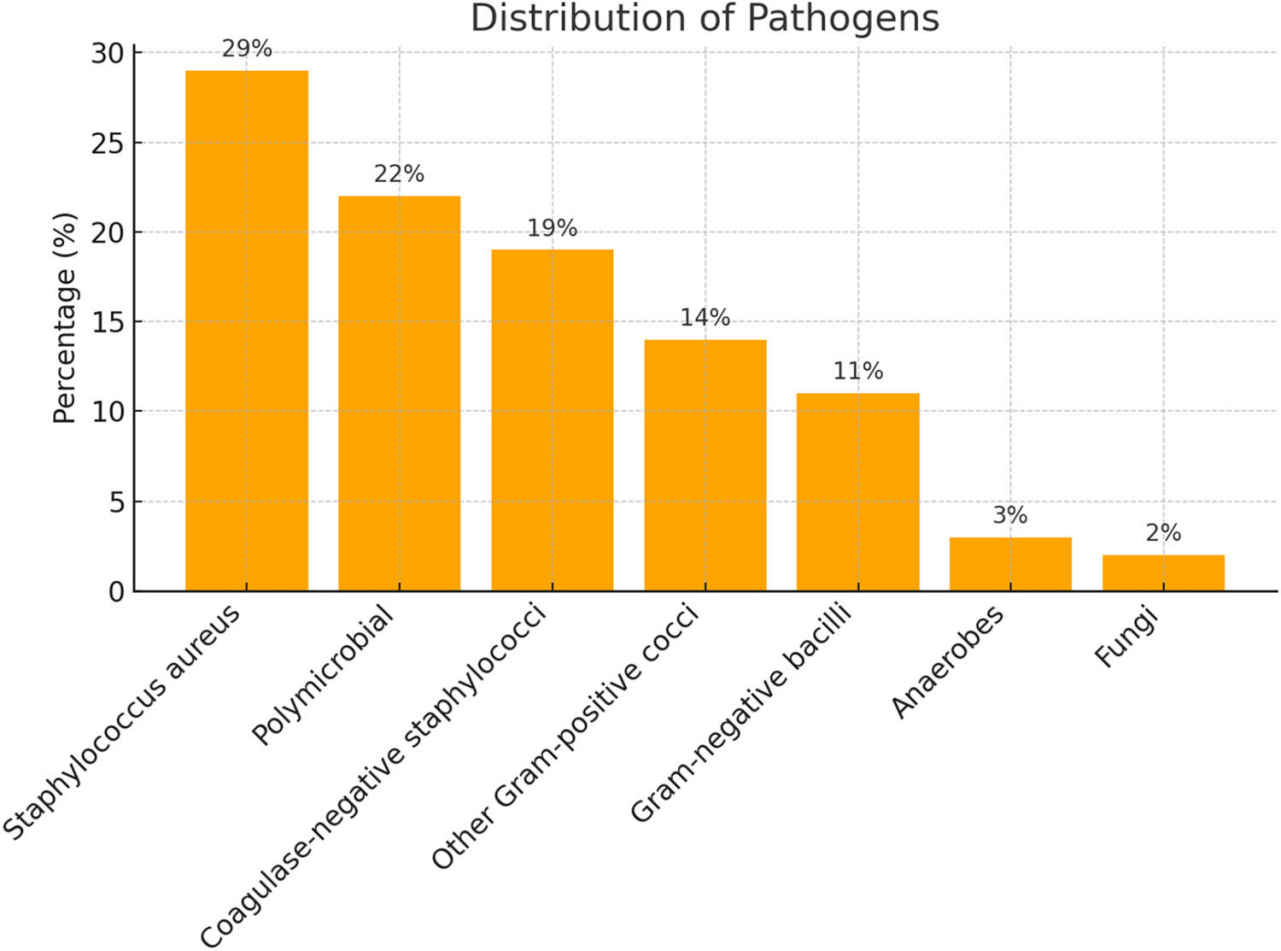
Microbiological results of the TKA/F– group. TKA, total knee arthroplasty.

Kaplan–Meier analysis demonstrated no significant difference in infection‐free survival between TKA/F+ and TKA/F– groups (log‐rank test, *p* = NS; Figure 4). Survival curves were truncated at 70 months due to the limited number of patients at risk beyond this time point.

**Figure 4:**
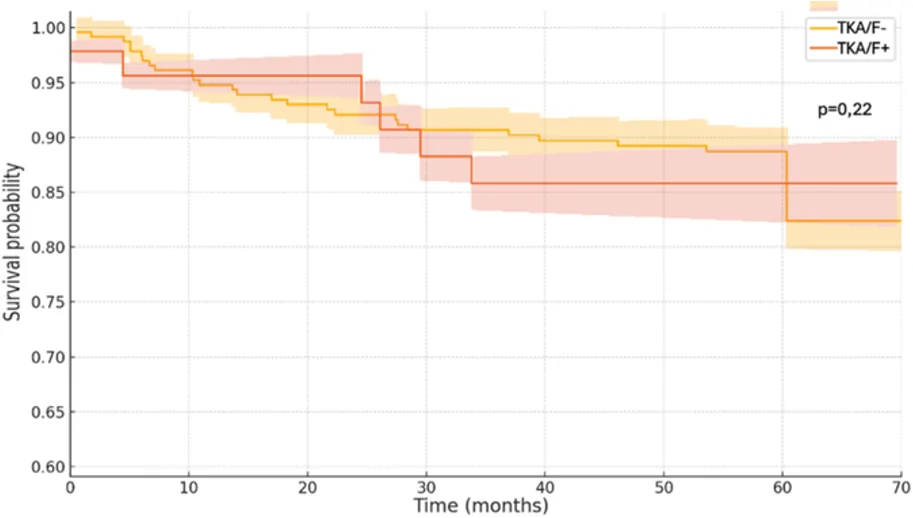
Comparison of Kaplan–Meier survival curves using the log‐rank test. TKA, total knee arthroplasty.

Among treatment failures in the TKA/F+ group, most episodes corresponded to new infections caused by microorganisms different from those identified at index surgery, whereas relapses with identical pathogens were less frequent. In contrast, failures in the TKA/F– group were predominantly due to microbiological relapse.

Soft‐tissue reconstruction using a local or free flap was required in 17 patients (36%) in the TKA/F+ group, with no flap‐related complications reported.

In multivariable logistic regression analysis, the presence of a sinus tract was not independently associated with treatment failure. Polymicrobial infection and higher ASA score were identified as independent predictors of failure, whereas age, BMI and diabetes were not significantly associated with outcome after adjustment.

Because of the imbalance between groups, a post‐hoc power analysis revealed limited ability to detect small differences in infection‐free survival, indicating a potential risk of Type II error. Nevertheless, this cohort represents one of the largest series of fistulized chronic infected TKAs treated with a single‐stage strategy in a referral centre setting.

## DISCUSSION

The most important finding of the present study is that the presence of a sinus tract was not associated with a lower infection‐free survival after single‐stage revision TKA. Despite a significantly higher medical burden and more complex microbiological profiles in fistulized patients, infection eradication rates were comparable between fistulized and non‐fistulized chronic PJIs. These findings challenge the traditional view that a sinus tract constitutes a contraindication to single‐stage revision in infected TKAs. This paradigm shift is in line with recent calls to reconsider overly restrictive contraindications in the management of PJI [6].

Patients in the TKA/F+ group presented with more unfavourable baseline characteristics, including older age, higher BMI, higher ASA scores and a greater prevalence of diabetes. From a microbiological standpoint, polymicrobial infections were significantly more frequent in this group, a factor known to negatively influence treatment outcomes [14]. Nevertheless, sinus tract presence was not independently associated with treatment failure in multivariable analysis. To our knowledge, this study represents the first comparative investigation specifically evaluating single‐stage revision outcomes in fistulized versus non‐fistulized infected TKAs using infection‐free survival as the primary endpoint.

Our results are consistent with previously published outcomes of single‐stage revision in septic knee arthroplasty [24]. Similar infection‐free survival rates after revision TKA for PJI have recently been reported in large single‐centre cohorts, even in complex and comorbid populations [13]. In an unselected cohort, Jenny et al. reported a failure rate of 17% following single‐stage exchange, although the proportion of fistulized cases was not specified [7]. Other series have described higher success rates, but most were limited by small sample sizes, retrospective designs, and heterogeneous inclusion criteria. Zahar et al. reported a 93% infection‐free survival at 10 years using rotating‐hinge implants, enabling extensive soft‐tissue resection including collateral ligament planes [30]. Haddad et al. reported a 100% success rate, but only included 28 highly selected patients without soft‐tissue compromise or polymicrobial infection and with known, antibiotic‐sensitive organisms [5].

The wide variability in reported infection control rates for single‐stage revision (ranging from 75% to 100%) likely reflects substantial differences in patient selection, surgical protocols, antibiotic strategies and follow‐up duration. These limitations have been highlighted in several systematic reviews [9, 16, 17, 24]. While some studies suggest that single‐stage revision may offer superior outcomes compared to two‐stage procedures [16], these results should be interpreted cautiously due to methodological heterogeneity.

The multicenter comparative study conducted by the French Society for Hip and Knee Surgery (SFHG) specifically addressed the role of patient selection [7]. No significant difference in eradication rates was observed between strictly selected patients and unselected patients after four years of follow‐up (78% vs. 85%, respectively). These findings support the notion that rigid exclusion criteria may not be mandatory when standardized multidisciplinary protocols are applied.

In the present study, favourable outcomes can likely be attributed to a rigorous and standardized surgical and medical strategy. Surgical management systematically included complete removal of foreign material, aggressive soft‐tissue debridement, extensive irrigation and systematic deep tissue sampling. Fixation was performed using gentamicin‐loaded cement, which has been shown to reduce the risk of subsequent infection following one‐stage exchange [8]. Recent registry data have further confirmed the protective effect of antibiotic‐loaded bone cement on the risk of septic revision in TKA [4]. When primary wound closure was not achievable, soft‐tissue reconstruction using local pedicled or free flaps was performed, most commonly using the medial gastrocnemius muscle, a technique well recognized for reducing reinfection rates in infected TKAs [22].

Antibiotic management was standardized and coordinated by the CRIOAC multidisciplinary team. Broad‐spectrum empirical intravenous therapy was initiated postoperatively and secondarily adapted according to microbiological results, in accordance with national recommendations [11]. Although preoperative antibiotic prophylaxis was administered, prior studies have demonstrated that this practice does not compromise intraoperative culture yield [27].

Several limitations of this study should be acknowledged. First, its retrospective and single‐centre design may limit generalizability. However, the study was conducted in a regional referral centre for complex bone and joint infections, which may mitigate variability in surgical expertise and follow‐up protocols. Second, baseline differences between groups reflect the greater frailty of fistulized patients, a phenomenon previously described in patients with orthopaedic device‐related infections [29]. Third, the analysis focused exclusively on septic outcomes, without evaluation of functional or patient‐reported outcomes. Fourth, long‐term survival analysis beyond 70 months was not reliable due to the increasing proportion of censored data.

An additional limitation relates to the limited statistical power of the study. Owing to the relatively small number of fistulized cases, the study was underpowered to detect small differences between groups, and a type II error cannot be excluded. Nonetheless, given the rarity of fistulized chronic TKA infections and the long inclusion period, this cohort remains clinically meaningful.

From a clinical perspective, these results suggest that the presence of a sinus tract alone should no longer be considered an absolute contraindication to single‐stage revision in chronic infected TKAs, particularly when management is performed in experienced referral centres with standardized multidisciplinary protocols. This approach may offer substantial advantages by avoiding the morbidity, prolonged immobilization and socioeconomic burden associated with two‐stage procedures.

## CONCLUSION

There was no significant difference in infection‐free survival between fistulized and non‐fistulized groups undergoing single‐stage TKA revision. Our working hypothesis is therefore supported: the presence of a sinus tract does not appear to be a contraindication to single‐stage management of infected TKAs.

The cornerstones of successful single‐stage treatment remain meticulous surgery with thorough soft tissue debridement, extensive irrigation, appropriately targeted intravenous antibiotic therapy (initially empirical and subsequently adapted), administered for a minimum duration of three months and durable wound closure—potentially requiring a local flap when necessary.

## AUTHOR CONTRIBUTIONS

Arthur Barbaret wrote the manuscript. Patrice Wong and David Zhu made data curation. Joseph Jacquet, Louis Lajoinie and Clement Ferri made investigations. Florian Collignon and Kamel Rouizi made conceptualization. Thomas Oberle and Gregoire Vaz made bibliography research. Sibylle Bevilacqua, Andrea Fernandez and Olivier Roche reviewed the manuscript.

## CONFLICT OF INTEREST STATEMENT

Olivier Roche receives consulting fees from United Orthopedics. The remaining authors declare no conflict of interest.

## ETHICS STATEMENT

The local ethics committee of Centre Hospitalier Universitaire de Nancy (approval number 2024PI232) approved this single‐centre retrospective case‐control study. All patients received an opt‐out form that they had to return within a specified period if they did not wish to participate in the study.

## Data Availability

Data are available on request due to privacy/ethical restrictions.
